# Supplementary material for: Mating-Induced Trade-Offs upon Egg Production versus Fertilization and Offspring’s Survival in a Sawfly with Facultative Parthenogenesis
Source: Insects. 2021 Aug 2;12(8):693. doi: 10.3390/insects12080693 (PMC8396567; doi:10.3390/insects12080693)
Supplement: Supplementary file 1 [file insects-12-00693-s001.zip › Supplementary Files/Table S1-S3.pdf]

**Table S1** Primers for quantitative realtime PCR

| Gene ID | Forward Primer (5' to 3') | Reverse Primer (5' to 3') |
|---|---|---|
| *β-Tubulin* | CATCCAGAACAAGAACAGCTCG | ACGGGGAGGAATGTCACAAACA |
| *DN64818_c0_g6* | TGAATTGGGAAATCAGTAGCACTGTC | TGTTTGAGCCTTGCACACAAA |
| *DN70573_c2_g1* | AGTGACCACCCTTGCGATGC | TGCCTCCCATCAGACACCGA |
| *DN60391_c5_g2* | TGACGGTCTGGACCACCCAA | GCAGACTCGGAAGGCACCAT |
| *DN69705_c1_g3* | CCCTAAGGCGAGGCCGAAAG | GCTTCAGCACCGTAGTGCCT |
| *DN53654_c1_g7* | CATCCGGCGGTTTGTACTGC | CCGTGACCCGCAATCACTGT |
| *DN54310_c0_g6* | TCAAACAGACGCGCAAGGGA | TCGGGTCCAAACAGGCACAC |
| *DN65013_c0_g1* | TCCACTCCATACTGTTGGGATCA | AGTTTGGCTCCAGTTCAGTAAACA |
| *DN62024_c1_g1* | GGGAGACTGTACTGCGAAGC | TGCAAATCATTCAAGTTCCAACCA |
| *DN52313_c1_g5* | GTGCTCTTGGACACTCCCAT | CCGTGCCGTTGTTGAAAAGA |
| *DN65191_c2_g1* | GCGCTCGGACATGAATTTGC | CCACCACAGCCTGCTACAAG |
| *DN66978_c2_g1* | TCGTTGTAAGAAGCAGCTCGT | TGTCAGATGTCGCAGGATGGA |
| *DN78694_c0_g1* | ATTCGGCGATGGTTGAGTCG | TTGGGCTGGCGAATTTCACA |
| *DN63597_c1_g1* | GGAGCACCATGTGGCATACT | TTGCGAGAATGTGGTGGTGA |
| *DN62024_c1_g1* | GGGAGACTGTACTGCGAAGC | TGCAAATCATTCAAGTTCCAACCA |
| *DN65013_c0_g1* | TCCACTCCATACTGTTGGGATCA | AGTTTGGCTCCAGTTCAGTAAACA |
| *DN57206_c6_g2* | ATGAGCATCCGCCCTCTCCT | TTGTCCACCACGCTCACCAC |
| *DN71364_c1_g1* | TACCACGTTGGTCGACATGC | CCAGCTTGTGCTCCTGGATG |
| *DN70765_c7_g9* | GCTCCAGCATCCTTCGTAGC | GCCTTCCTCGGACAGGAGAT |
| *DN67787_c1_g2* | AACCGTCGGCATGGAGTGTG | GTGCAGAGGCAGCACAGGTT |
| *DN68243_c0_g3* | TGCACATTGAATGGCGCTTAGA | TCCCTTGAACTTCTCTCCAACAGT |
| *DN65710_c6_g4* | AAGCGAAAGAACGCGATGGT | TTCAACAGGTGAGCGCAGAG |
| *DN56824_c0_g2* | GATGCAGCCTGGATGCTGTT | CGCCCATCAACGTCACATCA |
| *DN51116_c1_g5* | GCGTACGACCCAAGCTTCAA | GTCCGTCAACGGTCTTCTGG |
| *DN45576_c0_g1* | TACGCCAGCACTGTTGACAC | TGGCAAGTTCAAGGCTGTCC |

**Table S2** Summary of the quality of all sample sequencing data

| #Sample_ID | Clean_Reads | A% | T% | C% | G% | N% | Error% | Q20% | Q30% | GC% |
|---|---|---|---|---|---|---|---|---|---|---|
| V1h-1 | 48732506 | 31.67 | 31.35 | 18.27 | 18.7 | 0 | 0.0248 | 97.97 | 94.54 | 36.97 |
| V1h-2 | 54865402 | 29.6 | 29.15 | 20.35 | 20.9 | 0 | 0.0251 | 97.78 | 94.27 | 41.25 |
| V6h-1 | 51945192 | 29.01 | 28.61 | 20.91 | 21.46 | 0 | 0.0248 | 97.89 | 94.53 | 42.38 |
| V6h-2 | 50279316 | 29.54 | 29.14 | 20.34 | 20.97 | 0 | 0.0247 | 97.99 | 94.66 | 41.32 |
| V24h-1 | 54548534 | 31.38 | 30.95 | 18.55 | 19.12 | 0 | 0.0248 | 97.9 | 94.51 | 37.67 |
| V24h-2 | 55232694 | 28.92 | 28.46 | 20.97 | 21.64 | 0 | 0.0247 | 97.93 | 94.62 | 42.61 |
| V24h-3 | 50962894 | 29.08 | 28.67 | 20.73 | 21.51 | 0 | 0.0248 | 97.89 | 94.53 | 42.24 |
| M1h-1 | 49009592 | 30.12 | 29.8 | 19.77 | 20.31 | 0 | 0.0252 | 97.76 | 94.1 | 40.08 |
| M1h-2 | 44700996 | 29.76 | 29.35 | 20.17 | 20.72 | 0 | 0.0247 | 97.94 | 94.64 | 40.89 |
| M1h-3 | 50686434 | 28.3 | 27.95 | 21.57 | 22.18 | 0 | 0.025 | 97.81 | 94.45 | 43.74 |
| M6h-1 | 47074780 | 30.48 | 30.08 | 19.37 | 20.06 | 0 | 0.0244 | 98.11 | 94.95 | 39.44 |
| M6h-2 | 46133412 | 30.42 | 29.96 | 19.45 | 20.16 | 0 | 0.0245 | 98.02 | 94.91 | 39.62 |
| M6h-3 | 51454412 | 30.08 | 29.61 | 19.85 | 20.46 | 0 | 0.0244 | 98.11 | 94.97 | 40.31 |
| M24h-1 | 47917526 | 30.76 | 30.35 | 19.08 | 19.81 | 0 | 0.0245 | 98.02 | 94.88 | 38.89 |
| M24h-2 | 48694016 | 30.86 | 30.51 | 19.04 | 19.59 | 0 | 0.0241 | 98.21 | 95.21 | 38.63 |
| M24h-3 | 47228838 | 28.93 | 28.57 | 20.97 | 21.53 | 0 | 0.0244 | 98.08 | 94.9 | 42.5 |

**Table S3** Pearson's correlation coefficient

| | V1h-1 | V1h-2 | V6h-1 | V6h-2 | V24h-1 | V24h-2 | V24h-3 | M1h-1 | M1h-2 | M1h-3 | M6h-1 | M6h-2 | M6h-3 | M24h-1 | M24h-2 | M24h-3 |
|---|---|---|---|---|---|---|---|---|---|---|---|---|---|---|---|---|
| V1h-1 | 1 | 0.923 | 0.955 | 0.878 | 0.767 | 0.846 | 0.796 | 0.885 | 0.892 | 0.802 | 0.959 | 0.806 | 0.876 | 0.841 | 0.854 | 0.865 |
| V1h-2 | 0.923 | 1 | 0.978 | 0.988 | 0.784 | 0.902 | 0.823 | 0.939 | 0.954 | 0.901 | 0.956 | 0.945 | 0.981 | 0.955 | 0.974 | 0.988 |
| V6h-1 | 0.955 | 0.978 | 1 | 0.967 | 0.776 | 0.892 | 0.803 | 0.921 | 0.936 | 0.871 | 0.977 | 0.906 | 0.961 | 0.91 | 0.936 | 0.961 |
| V6h-2 | 0.878 | 0.988 | 0.967 | 1 | 0.772 | 0.895 | 0.816 | 0.943 | 0.946 | 0.915 | 0.941 | 0.962 | 0.979 | 0.963 | 0.982 | 0.992 |
| V24h-1 | 0.767 | 0.784 | 0.776 | 0.772 | 1 | 0.962 | 0.983 | 0.921 | 0.912 | 0.816 | 0.762 | 0.768 | 0.772 | 0.863 | 0.824 | 0.76 |
| V24h-2 | 0.846 | 0.902 | 0.892 | 0.895 | 0.962 | 1 | 0.971 | 0.973 | 0.976 | 0.874 | 0.85 | 0.852 | 0.874 | 0.945 | 0.91 | 0.886 |
| V24h-3 | 0.796 | 0.823 | 0.803 | 0.816 | 0.983 | 0.971 | 1 | 0.945 | 0.942 | 0.845 | 0.795 | 0.811 | 0.812 | 0.906 | 0.863 | 0.802 |
| M1h-1 | 0.885 | 0.939 | 0.921 | 0.943 | 0.921 | 0.973 | 0.945 | 1 | 0.995 | 0.932 | 0.911 | 0.923 | 0.934 | 0.978 | 0.957 | 0.937 |
| M1h-2 | 0.892 | 0.954 | 0.936 | 0.946 | 0.912 | 0.976 | 0.942 | 0.995 | 1 | 0.925 | 0.919 | 0.916 | 0.935 | 0.98 | 0.958 | 0.942 |
| M1h-3 | 0.802 | 0.901 | 0.871 | 0.915 | 0.816 | 0.874 | 0.845 | 0.932 | 0.925 | 1 | 0.881 | 0.935 | 0.934 | 0.937 | 0.947 | 0.924 |
| M6h-1 | 0.959 | 0.956 | 0.977 | 0.941 | 0.762 | 0.85 | 0.795 | 0.911 | 0.919 | 0.881 | 1 | 0.928 | 0.963 | 0.894 | 0.936 | 0.943 |
| M6h-2 | 0.806 | 0.945 | 0.906 | 0.962 | 0.768 | 0.852 | 0.811 | 0.923 | 0.916 | 0.935 | 0.928 | 1 | 0.981 | 0.939 | 0.987 | 0.957 |
| M6h-3 | 0.876 | 0.981 | 0.961 | 0.979 | 0.772 | 0.874 | 0.812 | 0.934 | 0.935 | 0.934 | 0.963 | 0.981 | 1 | 0.945 | 0.987 | 0.983 |
| M24h-1 | 0.841 | 0.955 | 0.91 | 0.963 | 0.863 | 0.945 | 0.906 | 0.978 | 0.98 | 0.937 | 0.894 | 0.939 | 0.945 | 1 | 0.971 | 0.961 |
| M24h-2 | 0.854 | 0.974 | 0.936 | 0.982 | 0.824 | 0.91 | 0.863 | 0.957 | 0.958 | 0.947 | 0.936 | 0.987 | 0.987 | 0.971 | 1 | 0.977 |
| M24h-3 | 0.865 | 0.988 | 0.961 | 0.992 | 0.76 | 0.886 | 0.802 | 0.937 | 0.942 | 0.924 | 0.943 | 0.957 | 0.983 | 0.961 | 0.977 | 1 |
